# Global Surgery Priorities: A Response to Recent Commentaries

**DOI:** 10.15171/ijhpm.2019.10

**Published:** 2019-02-26

**Authors:** Jakub Gajewski, Ruairi Brugha, Leon Bijlmakers

**Affiliations:** ^1^ Institute of Global Surgery, Royal College of Surgeons in Dublin, Dublin, Ireland.; ^2^ Department of Epidemiology and Public Health Medicine, Royal College of Surgeons in Ireland, Dublin 2, Ireland.; ^3^ Radboud University Medical Centre, Nijmegen, The Netherlands.

We welcome the five published responses^1-5^ to our editorial,^6^ which outlined a research agenda for making surgery accessible in low- and middle-income country settings, where it is most needed. The commentators represent a good mix of academics, researchers and advocacy specialists, which demonstrates the growing global commitment to working together in the ‘empirically evolving global surgery systems science.’^3^ There is considerable consensus in the messages, including the importance of collaborative research approaches, adapted to country contexts; a focus on district population needs; and the use of standardised routine data collection and evaluation methods. Here, we briefly touch on some important new perspectives and some diverging ones.

Peck and Hannah helpfully emphasise the importance of Participatory Action Research (PAR) as a tool to gather and involve all relevant stakeholders in all stages of the research process.^7^ This is an important shift from the historical imposition of research agendas on local actors by researchers and research institutions from high-income countries.^8,9^ Our surgical systems strengthening research experience has shown the importance of working with national societies of surgical practitioners and representatives of Ministries of Health (MoHs) and Local Government in developing and implementing the research agenda. This is an essential step towards ensuring local relevance, structural support for effective scale-up of any health systems strengthening intervention, and long-term sustainability. It is local MoHs’ mandate and responsibility to improve health services in their countries. However, PAR involves challenges. Involvement of too many stakeholders with different interests and agendas can slow down implementation and disrupt or even derail an agreed research programme.

The commentaries diverge in the proposed approaches to coordinating the global surgery agenda in the pursuit of universal access to surgical, obstetric and anaesthesia care. Makasa^1^ and Henry^2^ suggest a globally coordinated approach, with international organisations defining directions for implementers to follow. While we acknowledge the importance of global coordination – uncoordinated parallel initiatives can overwhelm countries, from national to district level^10^ – we propose a demand-driven approach, enabling local stakeholders to identify gaps and optimal solutions for the delivery of surgical services. In place of global coordination mechanisms, new initiatives need to place ‘southern’ partners (ministries) in the driving seat. Multiple global partners share responsibilities to ensure global coordination, which is being advanced at international conferences and meetings that bring together best practices from peer-reviewed published research and opportunities for cross-country learning. Most importantly, global initiatives need to ensure local relevance through engagement with country level stakeholders, who are in a position to lead on national and sub-national implementation of agreed activities. To achieve maximum impact, such interventions need to be designed and implemented as multi-country projects, to ensure cross-country learning; and use appropriate PAR methods^3^ and fit-for-purpose standardised tools and metrics.^2,3^

Scale-up, a term widely used in health systems strengthening, needs to become the end goal for any systems intervention, thus it needs an explicit methodology and dedicated resources. While not always available,^5^ external funding can help kick-start initial in-country interest, but must be accompanied by coordination between global and local actors, supporting and not taking over national government leadership of scale-up. Otherwise, external funding dependence at best results in one-off successes; and at worst in disengagement of local parties. Engagement of stakeholders starts with the recognition of local authorities, key players, champions and other ‘doers,’ committed to solving a given problem. Local participation in agreeing research and implementation methods and jointly gathering, analysing and interpreting findings is of equal importance. As Katz et al state: “*research and policies that are not grounded in implementation science may be limited to academic exercises.”* Also, interventions designed without an explicit scale-up strategy may not be sustained or replicated elsewhere, and therefore may not be incorporated into local policies. Scale-up strategies need to be embedded in the original project concept, which is why we reiterate the importance of country leadership. In Scaling up Safe Surgery for Rural populations (SURG-Africa - http://www.surgafrica.eu) we are seeking to adhere to these principles.

In the COST-Africa project (2011-2016) we developed a BSc course in general surgery for non-physician clinicians in Malawi. The team, comprising international researchers and national surgeons, drafted the training curriculum, embedded the course into the structures of the University of Malawi’s College of Medicine, and coordinated implementation and evaluation.^11^ The key element that led to sustainability was the leadership of the national surgeons, who had strong links with the national MoH. While the European Union funded project offered scholarships to the first cohort of trainees, two subsequent national cohorts are now being trained in one of Africa’s poorest countries, without external financial support.

Routine surgical data collection and monitoring systems are needed to assess surgical needs; monitor surgical outputs and identify gaps; define interventions and monitor implementation; and evaluate impact. Internationally comparable, national level data help in advocating for funds to bridge the gap between well- and less well-endowed countries,^2^ with a view to enhancing equitable access to surgical care. Indicators should be standardised, where possible, to facilitate cross-country comparisons and lesson-learning. However, the selection of data collection tools needs to be determined by the objectives, purpose and intended frequency of data collection. Henry proposes using the WHO SARA tool^12^ to collect multi-country facility-based data on surgical capacity (service availability and readiness). This makes sense if the purpose is to inform government plans for investing in surgical capacity.

In SURG-Africa, we reviewed a range of published surgical capacity assessment tools that are used internationally. We concluded that the most appropriate instrument in terms of ease of administration, time spent in the field, complexity of data processing and analysis, and feasibility of repeat use, was the Personnel, Infrastructure, Procedures, Equipment and Supplies tool, developed by Groen et al and used in several settings internationally.^13^ Its strength lies in its ability to produce a simple summary index score expressing surgical capacity in a numerical way, which allows comparisons over time and between countries. While the SARA tool also allows for cross-country and secular comparisons, it is more complex, not surgery-specific, and it requires more resources to administer, process and analyse the data - all of which militate against its periodic use for intervention monitoring. Debates like these, enabled by the *IJHPM*, help throw light on aspects of methodology.^3^

All commentators agree on the importance of district level facilities as a focus of global surgery. This is where the most common surgical cases including the bellwether procedures should be treated, as a step towards universal access to surgical care, while protecting higher-level referral hospitals from congestion. District surgery must have a prominent place in National Surgical, Obstetric and Anaesthesia Plans, and the district voice needs to be heard throughout National Surgical, Obstetric, and Anesthesia Plan (NSOAP) formulation and implementation. Peck and Hanna describe a bottom-up approach in Latin America to incorporating a community perspective on data collection and national surgical planning. In Africa, NSOAP development has often been “based on empirical observation and in-country experience by identified stakeholders.”^3^ However, the experience drawn on is usually that of specialist surgeons, who may have limited understanding of the reality of surgery at district hospitals. In many sub-Saharan African countries, district surgery is mainly or wholly provided by non-specialists, including general medical officers and non-physician clinicians.^14^ Although usually not part of surgical societies, they are capable to provide valuable input into national-level strategic planning. We endorse Katz et al’s call for an “integrated surgical ecosystem to bridge the gap between the boots on the ground and policy-makers.”^5^ Routine data on surgical output from operating district hospital theatre registers provide an essential and often neglected empirical base for NSOAPs, which specialist surgeons are best placed to interpret.

Peck and Hanna propose five core principles to inform the developing discipline of Global Surgery System Science.^3^ We endorse principles 1-3. Common surgery-specific terminology and metrics language are needed to facilitate a common understanding (Principle 1). Mixed methods, PAR methods are needed to evaluate surgical systems (Principle 2); and scientific rigour and development of research methodologies are needed as well (Principle 3). We agree that effective transnational teams are needed (Principle 4), but place more emphasis on the role of national surgical societies who need to be empowered and supported to translate the global surgery agenda into national strategies. We also propose qualifying Principle 5, in that the ‘learner’ is not necessarily a surgeon - which implies a specialist - who would need to evolve into a ‘systems aware global surgeon.’ In transitioning from COST- to SURG-Africa, we have learned to work with district surgical teams, which include a range of surgically active cadres (clinical officers, nurses and general doctors). Between them, if trained and supervised, they are well placed to provide a sustainable response to addressing district level surgical needs.^15^ Moreover, the term ‘global surgeons’ suggests specialists from high-income countries who undertake locums in African countries; but they may contribute little to building sustainable systems for rural populations to access essential elective and emergency surgery.^16^

National surgeons, often organised in a surgical society, are best placed to lead the national surgical system. External initiatives and global partnerships need to be aware of the consequences of engaging surgical specialists from low-resource countries into activities that take them away from the operating rooms in favour of non-surgical work. Globally driven strategic planning, project management and administration – in the form of consultancy work or positions with international agencies which are often well paid – may aggravate some of the problems that global initiatives try to solve, contributing to the shortage of surgical specialists at national and referral hospitals. Specialist surgical knowledge and skills *sensu stricto* are mainly relevant in the operating room, and for training and supervision of surgical trainees. Where appropriate, the specialists should be called upon to provide expert opinion about strategic directions and solutions proposed to address surgical systems constraints.

Implementation of the global surgery agenda should not only rely on global surgeons who are ‘desirous and capable of addressing the social responsibility of resolving the global surgical burden,’^3^ but on local experts who are appropriately trained, preferably in the region, and fit for the task. Others have noted that lack of engagement of research leaders in global surgery may result in lack of depth of scientific enquiry.^17^ When setting up global surgery projects of any scale, experts with formal training in research methods, design and statistical skills should be involved, as a source of knowledge about appropriate evaluation methods. There is also a need to define the set of skills, train experts other than surgeons and equip them with the required know-how on how to research, plan and administer surgical programmes, thereby building local capacity so as to reduce low- and middle-income country dependence on external technical support. Countries require trained health planners, human resource managers, financial specialists, and staff trained in public administration; as well as national and regional surgical specialist, generalist and non-physician surgical training programmes.^18^ We thank the *International Journal of Health Policy and Management* for facilitating a debate on next steps for building capacity and consensus on how to design and evaluate national strategies for scaling up surgery, not only in sub-Saharan Africa but across low- and middle-income countries.^6^

## Ethical issues

Not applicable.

## Competing interests

Authors declare that they have no competing interests.

## Authors’ contributions

All authors contributed substantively to all drafts. All authors read and approved the final manuscript. JG and RB conceived the concept of the paper. JG drafted the first sketch and led on the development of the manuscript with inputs from LB and RB.

## Authors’ affiliations

^1^Institute of Global Surgery, Royal College of Surgeons in Dublin, Dublin, Ireland. ^2^Department of Epidemiology and Public Health Medicine,Royal College of Surgeons in Ireland, Dublin 2, Ireland. ^3^Radboud University Medical Centre, Nijmegen, The Netherlands.
